# No Effect of Tart Cherry Juice or Pomegranate Juice on Recovery from Exercise-Induced Muscle Damage in Non-Resistance Trained Men

**DOI:** 10.3390/nu11071593

**Published:** 2019-07-14

**Authors:** Kirstie L. Lamb, Mayur K. Ranchordas, Elizabeth Johnson, Jessica Denning, Faye Downing, Anthony Lynn

**Affiliations:** 1 Food and Nutrition Group, Sheffield Hallam University, Sheffield S1 1WB, UK; 2 Academy of Sport and Physical Activity, Sheffield Hallam University, Sheffield S10 2BP, UK; 3 Department of Oncology, Human Nutrition, University of Sheffield, Sheffield S10 2TN, UK

**Keywords:** exercise-induced muscle damage, recovery, polyphenols, tart cherry, pomegranate

## Abstract

Tart cherry juice (TC) and pomegranate juice (POM) have been demonstrated to reduce symptoms of exercise-induced muscle damage (EIMD), but their effectiveness has not been compared. This randomized, double-blind, parallel study compared the effects of TC and POM on markers of EIMD. Thirty-six non-resistance trained men (age 24.0 (Interquartile Range (IQR) 22.0, 33.0) years, body mass index (BMI) 25.6 ± 4.0 kg·m^−2^) were randomly allocated to consume 2 × 250 mL of: TC, POM, or an energy-matched fruit-flavored placebo drink twice daily for nine days. On day 5, participants undertook eccentric exercise of the elbow flexors of their non-dominant arm. Pre-exercise, immediately post-exercise, and at 24 h, 48 h, 72 h and 96 h post-exercise, maximal isometric voluntary contraction (MIVC), delayed onset muscle soreness (DOMS), creatine kinase (CK), and range of motion (ROM) were measured. The exercise protocol induced significant decreases in MIVC (*p* < 0.001; max decrease of 26.8%, 24 h post-exercise) and ROM (*p* = 0.001; max decrease of 6.8%, 72 h post-exercise) and significant increases in CK (*p* = 0.03; max increase 1385 U·L^−1^, 96 h post-exercise) and DOMS (*p* < 0.001; max increase of 26.9 mm, 48 h post-exercise). However, there were no statistically significant differences between treatment groups (main effect of group *p* > 0.05 or group x time interaction *p* > 0.05). These data suggest that in non-resistance trained men, neither TC nor POM enhance recovery from high-force eccentric exercise of the elbow flexors.

## 1. Introduction

Eccentric muscle contractions, which are a feature of many sports, can induce substantial muscle damage [1]. Typical symptoms of exercise-induced muscle damage (EIMD) include loss of force, a reduced range of motion (ROM), and the development of muscle soreness. Since these symptoms can impair subsequent performance, there is much interest in strategies that accelerate recovery from EIMD [1,2,3]. The development of EIMD is not fully understood, but it has been described as a two-phase process [2]. The initial phase is believed to involve structural disruption to sarcomeres and failure of the excitation-coupling process [4,5]. The secondary phase is characterized by a large increase in intracellular Ca^2+^, which: (i) activates calcium-dependent proteases that degrade muscle proteins [6], and (ii) triggers an inflammatory response [7]; a feature of these is an elevation in reactive oxygen species [8]. Since inflammation and reactive species are involved in the process of EIMD, numerous dietary factors with antioxidant and anti-inflammatory effects have been examined to determine whether they accelerate recovery from EIMD [3]. 

Polyphenols are one dietary component with antioxidant and anti-inflammatory effects that have recently attracted substantial interest for their potential to promote recovery from EIMD [1]. Studies have explored the effects of isolated polyphenolic supplements such as quercetin [9], and various foods and drinks rich in polyphenols including beetroot [10], bilberry [11], blueberry [12], cocoa flavonoids [13], green tea extract [14], pomegranate [15], and tart cherry [16]. The results of these studies have been somewhat equivocal. This could reflect differences in study design, but also probably reflects differences in the total content and profile of polyphenols that are present in the dietary supplements employed in the various studies. Different plant foods and different cultivars of the same fruit or vegetable can vary substantially in their content of polyphenols [17,18]. Moreover, even the same cultivar of a fruit or vegetable can vary manifold in its content of polyphenols depending on factors such as growing conditions and ripeness [19]. 

Of the various polyphenol-rich foods studied so far, tart cherries and pomegranate have arguably shown the most potential for promoting recovery from EIMD. Connolly et al. [16] were the first to report that tart cherry juice (TC) could accelerate recovery from EIMD. In untrained young men, the consumption of two × 12 fl oz bottles of a tart cherry/apple juice blend daily for 4 days prior to and four days after a bout of maximal eccentric contractions of the elbow flexors (2 × 20 reps) reduced post-exercise muscle soreness and reduced the loss in isometric strength. Since then, tart cherry juice has been shown to promote the recovery of muscle strength/function after: (i) a marathon [20], (ii) an eccentric knee extensor protocol [21], (iii) cycling [22], and (iv) high-intensity shuttle running [23,24]. When exercise protocols have induced a significant increase in inflammation, tart cherry has typically attenuated the rise in one or more serum markers of inflammation [20,22,23,25,26]. Positive effects on markers of oxidative stress [20,21,25] and DOMS [26,27] have also sometimes been observed, whereas tart cherry has demonstrated little ability to lower serum markers of muscle damage, such as creatine kinase [20,21]. Although studies on tart cherry have typically reported beneficial effects on one or more markers of EIMD, a couple of studies have failed to report any beneficial effects [28,29]. McCormick et al. [28] found no effect of supplementation with tart cherry juice on DOMS and markers of inflammation and oxidative stress in male water polo players who undertook a simulation designed to mimic the demands of a game of water polo. Beals et al. [29] failed to find a beneficial effect of supplementation with a beverage containing freeze-dried tart cherry powder on the recovery of muscle strength or DOMS in recreationally active men and women who completed a fatiguing eccentric knee flexor protocol. However, in that study the exercise protocol failed to induce measurable muscle damage. 

To our knowledge, four studies have reported on the ability of pomegranate to accelerate recovery from EIMD. In an initial cross-over study of 16 recreationally active young men, Trombold et al. (2010) reported that a daily supplement of pomegranate-extract taken for nine days was effective at enhancing the recovery of isometric strength after a bout of maximal eccentric contractions of elbow flexors (2 × 20 reps) undertaken on day 5 [15]. However, they found no difference between pomegranate extract and placebo for reported muscle soreness or serum markers of muscle damage and inflammation. In a subsequent study of resistance trained men, Trombold et al. (2011) found that a daily supplement of pomegranate juice (POM; taken for eight days before and seven days after a muscle damage protocol) enhanced the recovery of isometric strength of the elbow flexors. However, POM was ineffective at promoting the recovery of isometric strength of the knee extensors [30]. Then, the same group conducted a dose–response study in recreationally active young men, and found that one or two daily drinks of a POM concentrate were similarly effective at promoting the recovery of isometric strength of knee extensors following downhill running and of elbow flexors after 40 eccentric contractions [31]. In a small study of nine elite weightlifters, Ammar et al. (2016) reported that supplementation with POM for two days before a session of three Olympic weightlifting exercises seemed to have some beneficial effects on serum markers of muscle damage and reported muscle soreness measured acutely post-exercise and 48 h later [32]. However, the data are difficult to interpret, because all the participants consumed the placebo drink first and the two exercise sessions were separated by only 48 h. So, it seems that during the 48-h recovery period after the placebo drink, participants were consuming POM. 

Collectively, these studies indicate that TC and POM seem to accelerate post-exercise recovery; however, it is unclear whether they are equally as effective. Although POM and TC are both rich sources of anthocyanins, especially cyanidin and its derivatives [33], their profile of other polyphenols differs. For example, POM is particularly rich in ellagitannins, especially punicalagin and its isomers [34]. Whereas tart cherry contains flavan-3-ols such as catechin and epicatechins [35], which are absent from POM [34]. Due to these and other differences in composition, the potency of TC and POM to enhance recovery may differ, but no studies have directly addressed this possibility. This is surprising, because it is of practical importance to know whether the efficacy of TC and POM varies, so that optimal advice can be provided to athletes. Therefore, the purpose of this study was to compare the efficacy of TC and POM to promote recovery from EIMD.

## 2. Materials and Methods

### 2.1. Participants

Participants were recruited via word of mouth, poster advertisements in Sheffield Hallam University and the University of Sheffield, and recruitment emails to staff and students. Thirty-six non-resistance trained men (age 24.0 (interquartile range (IQR) 22.0, 33.0) years, body mass index (BMI) 25.6 ± 4.0 kg·m^−2^) volunteered to participate. Inclusion criteria included aged 18–65 years, non-smokers, no upper-body strength training in the last three months, injury-free, and not presently taking anti-inflammatories or antioxidants. The study was conducted in accordance with the Declaration of Helsinki. Ethical approval was received from Sheffield Hallam University Research Ethics Committee (SBS-244). Following written and verbal study briefings, participants provided written informed consent.

### 2.2. Study Design

**Figure 1:**
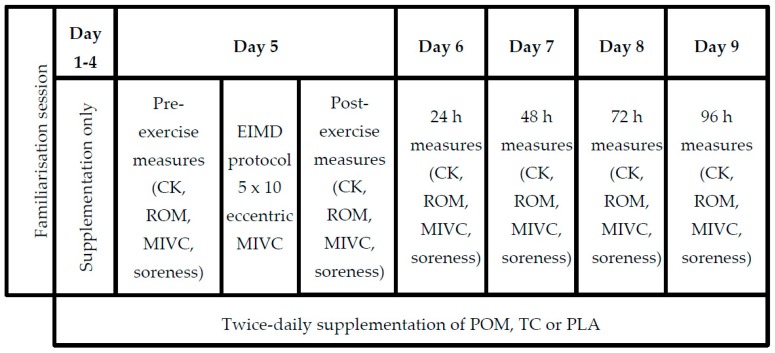
Timeline of study protocol from familiarization to 96-h post-exercise. POM = pomegranate; TC = tart cherry; PLA = placebo; EIMD = exercise-induced muscle damage; CK = creatine kinase; ROM = range of motion; MIVC = maximal isometric voluntary contraction.

This was a nine-day, randomized, double-blind, placebo-controlled, parallel study with three experimental treatment groups. Participants were block randomized to receive either: (i) pomegranate juice (POM), (ii) tart cherry juice (TC), or (iii) a fruit-flavored placebo drink (PLA). The order of the blocks (sizes 3 and 6) was determined using a computerized random number generator (www.random.org) by a researcher not involved in participant recruitment. At the start of the study, there was a familiarization session during which baseline measures of body composition were collected and participants practiced the protocols for inducing muscle damage and measuring strength (maximal voluntary isometric contractions (MIVC)). Participants were instructed to use minimal effort to prevent unintentional muscle damage and to ensure they did not induce a protective repeated bout effect [36]. Participants consumed 2 × 250 mL servings per day (one in the morning and one in the evening) of their allotted drink for nine days. On day 5, participants attended the research laboratory after consuming their normal breakfast and their morning test drink. Pre-exercise measures of MIVC, range of motion (ROM), delayed onset muscle soreness (DOMS), and blood creatine kinase (CK) were collected; then, participants completed a muscle damage protocol consisting of 5 × 10 sets of unilateral eccentric elbow flexions using their non-dominant arm. Participants consumed an extra 250 mL of their allotted drink and consumed a standardized meal immediately post exercise. The consumption of other food or drink, except water, was avoided for 3 h post-exercise. Further measures of MIVC, ROM, DOMS, and CK were collected immediately post-exercise and daily for four days (Figure 1). For the duration of the study, participants were asked to avoid: strenuous physical activity, other methods for promoting recovery from EIMD (including non-steroidal anti-inflammatory drugs, foods rich in polyphenols, and nutritional supplements). The physical activity diaries were used to check compliance with the instructions to avoid strenuous exercise, and the food diary was used to calculate the average number of portions of polyphenol-rich foods consumed daily across the duration of the study.

### 2.3. Fruit Juices, Placebo Beverage and Post-Exercise Meal

Each serving of POM (POM Wonderful LLC, Los Angeles, CA, USA) consisted of 250 mL of undiluted juice, whilst a serving of TC (CherryActive Ltd., Hanworth, UK) contained 30 mL of concentrate diluted with 220 mL of water. The energy content of the TC was adjusted to match the energy content of POM by the addition of maltodextrin powder (My Protein Ltd., Northwhich, UK). The PLA beverage consisted of a blackcurrant-flavored maltodextrin sports drink (Science in Sport Energy Go, SiS Ltd., Nelson, Lancashire, UK), which was prepared to match the energy content of the POM and TC drinks. The serving sizes of POM and TC were based on standard serving sizes and were congruent with volumes shown to be effective in previous studies [21,25,30]. A technician not involved in data collection prepared all the drinks in brown bottles to mask the color from the investigators. At recruitment, participants were informed that the aim of the study was to explore the effect of different fruit-flavored drinks on EIMD, so participants were unaware of which drink was the placebo drink. Fresh drinks were prepared and delivered to participants every two days. Participants were advised to store the drinks in their refrigerators to minimize the degradation of bioactive compounds. Empty bottles were counted to assess compliance.

The total phenolic content of the test drinks was analyzed using the Folin–Ciocalteu method, and results were expressed as gallic acid equivalents [37]. The pH differential method was employed to determine the total content of anthocyanins present in each drink, and results were expressed as cyanidin-3-glucoside equivalents [38]. For both analyses, three bottles of each juice were analyzed in triplicate. 

The post-exercise meal consisted of two white-bread rolls (8 g protein·100 g^−1^; Kingsmeal, Maidenhead, UK) containing chicken breast (30.7 g protein·100 g^−1^; Sainsbury’s Supermarkets Ltd., London, UK) or mature cheddar cheese for vegetarians (25.4 g protein·100 g^−1^; Cathedral City, Dairy Crest, Surrey, UK) with soya spread (Pure, Kerry Foods, Warrington, UK). Sandwiches were prepared to provide a total of 0.3 g protein·kg BW^−1^.

### 2.4. Anthropometric Measures

Height was measured to the nearest 0.1 cm using a wall-mounted stadiometer (Harpenden, Holtain Model 602VR, UK). Weight and body composition were measured using an InBody 720 bioelectrical impedance scale (Biospace Co., Ltd., Leicester, UK).

### 2.5. Measurement of Maximal Isometric Voluntary Contractions (MIVC)

At the familiarization session, the Biodex System3 isokinetic dynamometer (Biodex Medical Systems, Inc., Shirley, NY, USA) was set up according to manufacturer settings [39] for elbow flexion with the dynamometer orientated at a 30° angle and the position of the seat adjusted for each individual. Settings were recorded to ensure that participants were in the same position for each test. To measure maximal isometric strength, participants completed 5 × 3 s MIVC at a 90° angle with a 10-s rest interval between repetitions. The mean of the five repetitions was recorded. 

### 2.6. Eccentric (EIMD) Protocol 

A two-minute rest period was provided between measuring MIVC and the EIMD protocol. The eccentric exercise protocol consisted of 50 (five sets of 10) maximal voluntary eccentric contractions of the non-dominant elbow flexors on the isokinetic dynamometer. With the wrist supinated, the elbow joint was extended forcibly through 90°, from 90° to 180°, at an angular velocity of 60°·s^−1^. To ensure maximal resistance throughout each extension, verbal encouragement was given. The eccentric contraction was followed by a 12-s rest period during which the arm was returned to the flexed 90° position at 10°·s^−1^. A two-minute rest period was provided between sets. 

### 2.7. Measurement of Creatine Kinase 

Blood samples were obtained via finger prick capillary sampling. A 30-μL sample of capillary blood was applied to a CK test strip (Reflotron*^®^*, HaB Direct, Warwickshire, UK). Blood samples were analyzed for CK concentration using a Roche Reflotron*^®^* Plus analyser (HaB Direct, Warwickshire, UK). When CK values exceeded the measurement range of the analyzer, samples were diluted with a control blood sample of a known low CK concentration and reanalyzed. To calculate the CK concentration of these samples, the reanalyzed values were multiplied by the dilution factor, and the concentration of CK in the control was subtracted. A hyper-response was defined as a CK measurement above 2000 U·L^−1^ at any time-point following the EIMD protocol [36,40].

### 2.8. Measurement of Range of Motion and Elbow Flexion Soreness 

Maximal elbow extension and flexion of the non-dominant arm were measured using a 12-cm goniometer (True Angle Goniometer, Bodycare, Warwickshire, UK), and the total ROM was calculated. With the participant standing, the goniometer axis was placed on the pre-marked lateral epicondyle of the elbow. One arm of the goniometer was lined up with the humerus and acromion process at a pre-marked point. The other was placed in line with the distal radius, parallel to the forearm. To maintain consistency between measures, these points were marked with permanent ink at the beginning of study.

Elbow flexor soreness was rated on a 100-mm visual analogue scale (VAS) that was anchored with “no pain” at the left end of the scale (score of 0) and “unbearable pain” at the right end (score of 100). Participants were asked to complete two bicep curls with their non-dominant arm using a 1-kg dumbell, and then indicate on the scale the intensity of soreness experienced. 

### 2.9. Statistical Analysis

Descriptive characteristics at baseline were compared across treatment groups using one-way analysis of variance (ANOVA) tests or Kruskall–Wallis tests as appropriate. The effect of the intervention on MIVC, ROM, CK, and DOMS was assessed using mixed model ANOVAs (time as the within-group factor and treatment as the between group factor). Post-exercise data points for MIVC and ROM data were converted to the percentage of their respective baseline values before data analysis. When data failed Mauchley’s test of sphericity, the degrees of freedom where corrected using Greenhouse–Geisser estimates. Statistically significant main effects of time were explored using Bonferroni post-hoc tests. The MIVC, ROM, CK, and DOMS data were analyzed with and without the participants that were classified as hyper-responders based on a post-exercise CK value >2000 U·L^−1^ All the analyses were conducted on IBM SPSS statistics version 24.0 (SPSS Inc., Chicago, IL, USA), and *p* < 0.05 was set as the critical level of significance.

## 3. Results

### 3.1. Analysis of POM and TC

Each serving of POM contained nearly three times more total phenolics than a serving of TC (878.9 ± 92.7 mg versus 294.7 ± 14.9 mg) and over six times more monomeric anthocyanins (49.4 ± 2.0 mg versus 7.7 ± 0.3 mg). 

### 3.2. Participants

The characteristics of participants in each diet group are displayed in Table 1. There were no statistically significant differences between groups for age (*p* = 0.97), BMI (*p* = 0.82), lean muscle mass (*p* = 0.86), baseline MIVC strength (*p* = 0.79), or baseline CK level (*p* = 0.63). All 36 participants completed the study. Of these, six participants (two TC, two POM, and two PLA) were classified as hyper-responders based on a post-exercise CK > 2000 U·L^−1^. Data is missing for one participant at 72 h (PLA) owing to illness. One CK value at 48 h (POM) and two MIVC values at 72 h (TC and POM) are missing because of technical difficulties. 

### 3.3. Compliance with Protocol

Participants reported consuming all their test drinks, but two participants reported that on one test day, they consumed both drinks in the evening after forgetting to consume a drink in the morning. There were no statistically significant differences in the portions of polyphenol-rich foods consumed by each diet group during the study (TC = 2.81 ± 1.19; POM =2.93 ± 2.23; PLA = 3. 97 ± 2.67, *p* = 0.44).

### 3.4. MIVC

**Figure 2:**
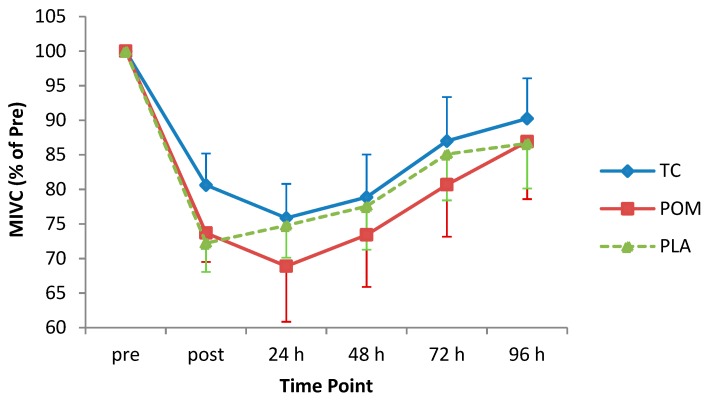
Change in maximal isometric elbow flexion strength following eccentric exercise (expressed as a percentage of pre-exercise strength). Values are an average of five maximal contractions and are displayed as mean (± SEM). Time by treatment, *p* = 0.91. MIVC = maximal isometric voluntary contraction; TC = tart cherry; POM = pomegranate; PLA = placebo, *n* = 11 for each group.

The muscle damage (MD) protocol induced a statistically significant reduction in MIVC (F_(2.9, 85.5)_ = 23.30, *p* < 0.001), which reached a nadir at 24 h post-exercise (max mean decrement 26.8%, Figure 2). Post-hoc Bonferroni analysis revealed statistically significant differences in strength from pre-exercise to: immediately post, 24 h post, 48 h post, and 72 h post-exercise (*p* < 0.05), but not 96 h post-exercise (*p* = 0.08). The reduction in MIVC over the complete post-exercise period was fairly similar in all groups ((F_(2, 30)_ = 0.27, *p* = 0.77; mean decrement TC = 14.6%, POM = 19.4%, and PLA = 17.3%), and the interaction between diet-group and time was not statistically significant (F_(5.7, 85.5)_ = 0.33, *p* = 0.91).

### 3.5. Elbow Flexor Soreness

**Figure 3:**
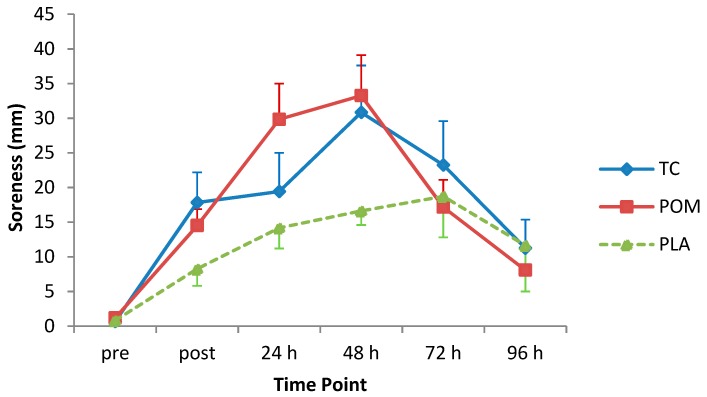
Change in soreness following eccentric exercise (reported on a scale of 0–100 mm). Values are displayed as mean (± SEM). Time by treatment, *p* = 0.10. TC = tart cherry; POM = pomegranate; PLA = placebo, *n* = 12 for TC, and POM and *n* = 11 for PLA.

There was a statistically significant increase in reported muscle soreness, which peaked 48 h (mean increase 26.9 mm) after the MD protocol (F_(3.2, 102.2)_ = 19.22, *p* < 0.001) (Figure 3). Post-hoc analysis showed that soreness scores were significantly different from baseline at all time-points after the MD protocol (*p* < 0.05). However, there was no statistically significant main effect of diet group (F_(2, 32)_ = 1.19, *p* = 0.32; mean increase in soreness: TC = 17.2 mm; POM = 17.4 mm; PLA = 11.7 mm) or diet group by time interaction (F_(6.4, 102.2)_ = 1.81, *p* = 0.10).

### 3.6. ROM

**Figure 4:**
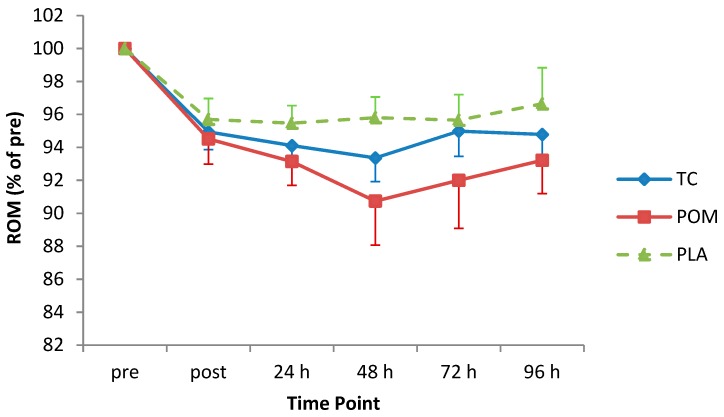
Range of motion (ROM) following eccentric exercise (expressed as a percentage of pre-exercise ROM). Values are displayed as mean (± SEM). Time by treatment, *p* = 0.71. TC = tart cherry; POM = pomegranate; PLA = placebo, *n* = 12 for TC and POM; and *n* = 11 for PLA.

There was a significant decrease in the ROM of the elbow flexors after the MD protocol (main effect of time: F_(1,6)_ = 11.60, *p* < 0.001; max mean decrement 6.8% at 48 h) (Figure 4). Post-hoc analysis showed that ROM was significantly lower at all post-exercise time-points in comparison to pre-exercise (*p* < 0.05). ROM did not differ significantly between diet groups (F_(1,3)_ = 1.23, *p* = 0.31; mean reduction in ROM: TC = 4.6%; POM = 6.1%; PLA = 3.5%), and there was no statistically significant interaction between diet group and time (F_(1,6;1,3)_ = 0.62, *p* = 0.71). 

### 3.7. Creatine Kinase Concentration 

**Figure 5:**
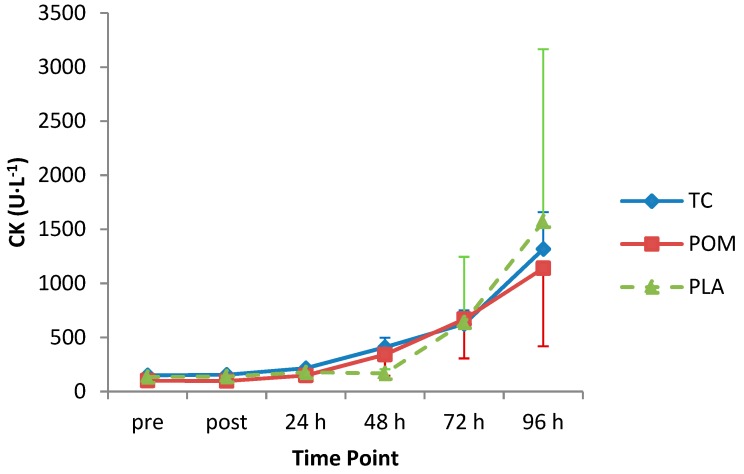
Creatine kinase (CK) following eccentric exercise. Values are displayed as mean (± SEM). Time by treatment, *p* = 0.93. TC = tart cherry; POM = pomegranate; PLA = placebo; n = 12 for TC, and n = 11 for POM and PLA.

Whole-blood CK increased after the MD protocol with peak values recorded 96 h post-exercise (1383 U·L^−1^; main effect of time: F _(1.1, 34.1)_ = 5.22; *p* = 0.03; Figure 5). There was no statistically significant main effect of diet group (F_(2, 31)_ = 0.05, *p* = 0.995; mean increase TC = 478.3 U·L^−1^; POM = 502.9 U·L^−1^; PLA = 471.6 U·L^−1^) or statistically significant interaction of diet group by time (F_(2.2, 34.1)_ = 0.09, *p* = 0.93). 

### 3.8. Exploratory Analysis with Hyper-Responders Removed

The dataset contained two participants in each group that were identified as hyper-responders on the basis that the concentration of CK in their blood rose above 2000 U·L^−1^ in the post-exercise period. An exploratory analysis omitting the data of these six hyper-responders did not reveal any statistically significant main effects of diet or diet × time interactions for any of the outcome markers (Table 2). 

## 4. Discussion

This is the first study to directly compare the effects of TC and POM on recovery from EIMD. We hypothesized that both juices would accelerate recovery relative to an energy-matched placebo drink. We also speculated that the potency of the juices might vary because of differences in their content of potentially anti-inflammatory and anti-oxidative phenolic compounds. However, we found that neither juice accelerated recovery as determined by changes in MIVC, DOMS, ROM, and CK, relative to an energy-matched placebo drink. Also, despite a compositional analysis revealing that a serving of POM contained almost three times more total phenolics and six times more monomeric anthocyanins than a serving of TC, these differences did not translate to any statistically significant differences between the effects of the two juices. 

The lack of effect of TC and POM on recovery of muscle strength was unexpected, because both drinks have consistently been reported to improve the recovery of MIVC [15,20,21,23,25,30,31]. To our knowledge, only one previous study has failed to report a beneficial effect of TC on recovery of strength when using a similar mode of resistance exercise [29]. However, in that study, the exercise protocol failed to invoke a substantial reduction in MIVC during the post-exercise period, thus making it impossible to detect a faster recovery of strength. In contrast, our MD protocol caused a statistically significant reduction in MIVC of a magnitude and time course that is consistent with studies that have reported beneficial effects of TC [20,21,23,25] or POM [15,30,31]. The lack of effect of TC and POM on MIVC was mirrored by a lack of effect on DOMS. Previous studies reporting the effect of TC and POM on DOMS have been inconsistent with approximately half of studies reporting a benefit [16,23,26,27,30,32,41], and half finding no treatment effects [15,20,21,22,24,29,31]. However, no study has reported a reduction in DOMS in the absence of a faster recovery of MIVC, so it is unsurprising that we found no effect of TC and POM on DOMS. 

Our MD protocol induced a significant elevation in blood CK, but there was no significant diet group or diet group by time interactions. The lack of effect of TC and POM on CK is consistent with existing literature [20,22,24,26,29]. CK levels started to rise substantially at 72 h post-exercise, and were at their highest at the 96-h time-point at the end of the study. This pattern of response is similar to other studies using a 50-repetition eccentric elbow flexion protocol in non-resistance trained men [15,42,43]. Consistent with the literature [44], we observed large inter-individual variations in the CK response to the MD protocol. This highly variable response between individuals and the fact that the magnitude of CK response does not necessarily correlate with the extent of muscle damage means that measuring CK responses may be of limited utility as a quantitative marker of muscle damage [45]. 

There was an approximate 7% decrease in relaxed elbow ROM in response to the MD protocol, which did not completely return to normal at 96 h. However, there was no difference in response between diet groups. To our knowledge, relaxed elbow ROM has only been measured in two studies investigating the effects of supplementation with TC [16,29], and no studies investigating the effect of supplementation with POM. In agreement with our study, TC did not alter the change in elbow ROM in previous studies [16,29]. 

A recent review on fruit-derived polyphenols and exercise reported that studies demonstrating favorable effects of TC on the recovery of muscle strength/function after a MD protocol typically supplied ≥1200 mg of total phenolics per day, and that two studies that failed to report a benefit may have done so because they supplied insufficient doses of TC-derived phenolics, which were 480 mg [27] and 733 mg [29] respectively [1]. The TC used in the current study contained approximately 300 mg of total phenolics per serving, resulting in a daily intake of approximately 600 mg. This might partly explain the lack of effect that we observed. The ability of TC to accelerate recovery and exert beneficial health effects is commonly attributed to its content of anthocyanins [46]. In some publications, the brand of TC used in the current study has been reported to contain 273 mg of total anthocyanins per 30-mL serving of concentrate [21,22]. However, using the pH differential method [38] to measure total (non-degraded) anthocyanins, we could only detect 7 mg of anthocyanins per 30-mL serving of concentrate. If intact anthocyanins make a substantial contribution to the protective effects of TC, then the low levels present in the TC could have contributed to its lack of effect. Anthocyanins are not particularly stable during processing and storage [47,48], so it is possible that the TC contained substantial quantities of anthocyanin derivatives that were not detected by our assay [38]. However, a low content of total phenolics and/or anthocyanins does not explain why the POM exerted no beneficial effects. The POM used in the current study provided slightly greater amounts of total phenolics (approximately 900 mg per serving) than reported in previous studies [15,30,31] and substantial amounts of anthocyanins (approximately 50 mg per serving). 

Previous studies that have investigated the effect of TC and POM to accelerate recovery from eccentric resistance exercise have most commonly used a crossover study design assigning contralateral limbs to each treatment. We used a parallel design, because we had three treatments. A parallel design has the advantage of removing the contralateral repeated bout effect that may influence the extent of damage in the limb tested second in a crossover study [49,50,51]. However, a disadvantage is that inter-individual variation in a parallel design dictates that large samples sizes are required to detect small and moderate effects. It is possible that we may have detected some treatment effects if we had used a larger sample size. However, others have reported significant effects of POM [31] and TC [20,24] on muscle strength or function using a parallel design with similar group sizes of between 10–15 participants, although in the case of TC with different types of damage protocols i.e., marathon running [20] and repeated sprints [24]. Moreover, in our study, there were no weak trends toward any treatment effects. Interrogation of our dataset revealed that two participants in each diet group exhibited a hyper-response to the MD protocol classified on the basis of a post-exercise CK > 2000 U·L^−1^. We conducted an exploratory analysis of the dataset with these ‘hyper-responders’ removed. This failed to reveal any statistically significant treatment effects and for most measures shifted the treatment outcomes further toward a null effect. 

This study has several limitations. It has been proposed that TC and POM may accelerate recovery from EIMD because of their antioxidant and anti-inflammatory effects [1]. In the present study, we did not measure any markers of oxidative stress or inflammation. It may have been informative to determine whether TC or POM reduced markers of oxidative stress such as F2-isoprostanes and protein carbonyls, with the caveat that changes measured in blood do not necessarily correlate with changes within muscles [52]. It would have been useful to document a reduction in exercise-induced inflammation after supplementation with TC and POM. However, common serum markers of inflammation such as c-reactive protein and interleukin-6 are typically not markedly elevated by muscle damage protocols based on single limbs [1]. We only measured one blood marker of muscle damage: creatine kinase. It is possible that measuring a larger number of markers may have revealed a treatment effect. It may have been illuminating to measure the appearance of polyphenolic metabolites in our participants after they consumed POM or TC. However, this would have required the repeated collection of blood and/or urine samples over a 24-h period. This was not feasible within the context of the current study design. Despite our instructions to avoid foods rich in polyphenols participants consumed some foods rich in polyphenols during the intervention. Although intake was similar in all three diet groups, it is possible that we may have detected an effect of POM or TC if participants had avoided all the foods containing polyphenols. However, this is almost impossible to achieve in a free-living study, because of the ubiquitous nature of foods and beverages rich in polyphenols. We compared serving sizes of TC and POM that have previously been reported to accelerate recovery from EIMD [21,25,30]. Our in-house analyses revealed that a typical serving of POM provided approximately three times greater amounts of total phenolics and approximately six times greater amounts of total anthocyanins than a typical serving of tart cherry concentrate. Matching the two drinks for total phenolic content may have provided a more equitable comparison, but it would have required participants to consume either six servings of TC concentrate per day or only a third of the volume of POM that has previously been reported to accelerate recovery [30].

## 5. Conclusions

The MD protocol used in the present study induced a substantial reduction in MIVC, a modest reduction in relaxed elbow ROM, a substantial elevation in blood CK, and a modest increase in reported muscle soreness. The TC and POM provided markedly different quantities of total phenolics and intact monomeric anthocyanins. However, there were no statistically significant differences in the effects of the two juices, and both juices failed to exert any statistically significant effect on the measured indices. The current results indicate that in non-resistance trained men neither TC nor POM enhance recovery from high force eccentric contractions of the elbow flexors. 

## Figures and Tables

**Table 1:** Participant characteristics (*n* = 36).

	Tart Cherry	Pomegranate	Placebo
*N*	12	12	12
Age (years)	24.0 (IQR 22.0, 33.0)	24.0 (IQR 21.0, 32.5)	24.0 (IQR 22.5, 32.0)
BMI (kg·m^−2^)	26.2 (± 3.9)	25.5 (± 3.9)	25.2 (± 4.5)
LMM (kg)	36.0 (± 5.2)	35.0 (± 6.1)	34.8 (± 6.2)
Baseline strength (N·cm^−2^)	41.8 (IQR 34.3, 53.2)	42.7 (IQR 33.6, 52.2)	44.1 (IQR 37.2, 51.5)
Baseline CK level (U·L^−1^)	148.9 (± 60.1)	150.3 (± 44.6)	130.9 (± 57.7)

Values are mean (± standard deviation (SD)) or median and interquartile range (IQR); BMI = body mass index; LMM = lean muscle mass; CK = creatine kinase.

**Table 2:** Analysis of outcome markers with hyper-responders removed.

Outcome Marker	Main Effect of Diet Group	Main Effect of Time	Diet × Time Interaction
MIVC	*p* = 0.78	*p* < 0.001	*p* = 0.97
DOMS	*p* = 0.28	*p* < 0.001	*p* = 0.26
ROM	*p* = 0.71	*p* < 0.001	*p* = 0.90
CK	*p* = 0.87	*p* = 0.01	*p* = 0.35

MIVC, maximal isometric voluntary contraction; DOMS, delayed onset of muscle soreness; ROM, range of motion; CK, creatine kinase.
